# ﻿Checklist and key to species of stink bugs (Hemiptera, Heteroptera, Pentatomidae) of Kentucky, United States of America

**DOI:** 10.3897/zookeys.1213.122843

**Published:** 2024-09-25

**Authors:** Armando Falcon-Brindis, Raul T. Villanueva

**Affiliations:** 1 Department of Entomology, Plant Pathology and Nematology, University of Idaho, Parma Research and Extension Center, Idaho, USA University of Idaho Idaho United States of America; 2 Department of Entomology, University of Kentucky, Research and Education Center, Kentucky, USA University of Kentucky Kentucky United States of America

**Keywords:** Economic importance, public databases, sampling biases, species diversity, taxonomy

## Abstract

Stink bugs (Heteroptera: Pentatomidae) have received a lot of attention as there are many economically important pest species. However, the status of species richness, distribution, and taxonomy remain overlooked and outdated in Kentucky (USA). Having such information at a regional scale is crucial to allow the development of suitable pest management and conservation programs. Here, the stink bug fauna of Kentucky was examined from museum specimens, literature, and public online repositories. Overall, 42 species in 28 genera and three subfamilies (Asopinae, Podopinae, and Pentatominae) are listed from Kentucky. Thirteen species are new records for Kentucky, 10 species are considered to be of economic importance and eight are strict predators. Pictures of species are provided along with the first key for the identification of the stink bug species of Kentucky.

## ﻿Introduction

The family Pentatomidae, also called stink bugs or turtle bugs, is the third most speciose family within the suborder Heteroptera, comprising 4949 species worldwide included in nine subfamilies (Asopinae, Cyrtocorinae, Discocephalinae, Edessinae, Pentatominae, Phyllocephalinae, Podopinae, Serbaninae, and Stirotarsinae), only surpassed by Reduviidae (~6000 species) and the Miridae (>11,000) (Schaefer and Panizzi 2000; Weirauch 2008; Cassis and Schuh 2012; Rider et al. 2018; Schuh and Weirauch 2020). Although stink bugs are more diverse in the Neotropical region (>1400 species) (Grazia et al. 2015), Nearctic pentatomids are probably among the best-studied insects in terms of diversity (~300 species), life history and distribution (Henry and Froeschner 1988, 1992). However, given the complexity of the group, both taxonomic and distributional changes are still emerging (Rider and Swanson 2021; Roca-Cusachs et al. 2022; Paim et al. 2022).

Adopting a regional approach to studying the pentatomid fauna provides a more accurate view of the group and facilitates the identification of species for either scientific or educational purposes (Packauskas 2012; Paiero et al. 2013). In this regard, the revision of Pentatomoidea provided by McPherson (1982) remains the most comprehensive taxonomic work in the northeastern United States. However, it emphasizes the fauna of Illinois and does not provide habitus illustrations for most of the species (*N* = 120), thus the identification process could be challenging. Recent efforts to update the stink bug fauna at the state scale include the pentatomids of Connecticut (O’Donnell and Schaefer 2012), Kansas (Packauskas 2012), Michigan (Swanson 2012), Minnesota (Koch et al. 2014), Missouri (Sites et al. 2012), New Mexico (Bundy 2012), North Dakota (Rider 2012), Ohio (Furth 1974), Virginia (Hoffman 1971), Washington (Zack et al. 2012), and the province of Ontario, Canada (Paiero et al. 2013).

The identification of most North American stink bugs can be reasonably conducted based on external morphology (some species groups can be challenging however, see Paim et al. 2022). Stink bugs are of moderate to large size, ranging from 4 to 20 mm in length, and generally ovoid or broadly elliptical in shape (Schuh and Weirauch 2020). Phytophagous stink bugs are usually more or less round in shape (some species associated with grasses are somewhat more elongated), usually with five-segmented antennae, three-segmented tarsi, and a subtriangular scutellum (Panizzi et al. 2000). In contrast, predatory stink bugs (subfamily Asopinae) are distinguished by an incrassate rostrum, particularly the first segment, which can swing forward fully, and the posterior margins of the buccula are merged (De Clercq 2008; Schuh and Weirauch 2020).

Pentatomids are considered an economically important group as most species are plant feeders (~90%), and about 10% prey upon arthropods, including many that are considered to be pests (McPherson and McPherson 2000; Schaefer and Panizzi 2000), in many agricultural systems (Panizzi and Slansky 1985; Pezzini et al. 2019). However, there are phytophagous species acting as facultative predators, but basic aspects of the biology of such species remain unknown, especially regarding behavior, population dynamics, and host damage (McPherson 1982). Moreover, stink bugs are associated with the transmission of plant pathogens causing boll rot, yeast spot, leaf spot, and different witch broom symptoms (Mitchell et al. 2018). Monitoring and identifying stink bugs in agricultural systems is critical for pest management purposes (Koch et al. 2014, 2017; Pezzini et al. 2019), especially when invasive species resembling native pentatomids are present. Several exotic species of economic importance reported in the United States can be easily misidentified and confused with native species without the help of suitable keys, e.g., the brown marmorated stink bug *Halyomorphahalys* Stål, the painted bug *Bagradahilaris* (Burmeister), and the southern green stink bug *Nezaraviridula* Linnaeus (Hoebeke and Carter 2003; McPherson 2018).

Color variation in adults can be a deceiving characteristic in species identification, for example, the predaceous species *Stiretrusanchorago* Fabricius displays contrasting bicolored and unicolored forms (Waddill and Shepard 1974). Species of the genus *Banasa* Stål and *Thyanta* Stål have seasonal green or brown forms (Thomas and Yonke 1981; Rider and Chapin 1992), and species of the *Euschistus* Dallas complex are often confused by the difficult taxonomic characters and multiple forms within a species (Paim et al. 2022). In addition, identifying immature stink bugs is a challenging task as there are not many studies on immature stages (DeCoursey and Allen 1968; Herring and Ashlock 1971; Brugnera et al. 2022).

Accurate identification of stink bugs at the regional scale is crucial to allow the development of suitable pest management and conservation programs (Grazia et al. 2015). Therefore, taxonomic keys supported by relevant information are valuable resources that help to avoid ambiguity and confusion in the identification of pentatomids. The purpose of this work is to provide an updated checklist and a key to the species of Pentatomidae occurring in Kentucky.

## ﻿Material and methods

The examined material was obtained from the University of Kentucky Insect Collection (UKIC), the Insect Collection of the University of Louisville (**ULIC**), and the University of Kentucky´s Research and Education Center (**UKREC**) at Princeton, KY. Additionally, species occurrence records of Pentatomidae were downloaded from the Global Biodiversity Information Facility (GBIF 2023) and the Symbiota Collections of Arthropods Network (SCAN 2023). These databases contained observation records from seven different sources, namely iNaturalist, Broward College, Monte L. Bean Life Science Museum, Oregon State Arthropod Collection (**OSAC**), Colorado State University (**CSU**), North Carolina State University (**NCSU**), Carnegie Museum of Natural History (**CMNH**), The Field Museum of Natural History, Illinois Natural History Survey (**INHS**) Insect Museum, and Texas A&M University (**TAMU**). Only research-grade observations from iNaturalist were carefully considered in this work, as many identifications of pentatomids can only be made by personal examination of specimens. Species synonyms are provided in this document following the literature indicated in Table 1. See Rider (2015) for more details about synonyms and previous combination names. After standard data cleaning (Chapman 2005), only records containing location and collection date were entered into a database to produce a distribution map and to create a plot of observations across time. Duplicate observations were removed from the final database.

**Table 1.** Checklist of species of Pentatomidae occurring in Kentucky. EI = Economic importance (marked with “x”). North America (NA) refers to Canada (CAN), United States (US), and Mexico (MX). Central America (CA), South America (SA). Cardinal directions are displayed in lower cases. Size is expressed as the length in millimeters. FH = Feeding habit. Phytophagous (Ph), Predator (Pr) and facultative predator (FaP). The source column indicates the sources used to identify the species. *New record for Kentucky State.

Taxon Synonym	EI	Distribution	Size	FH	Source
**Asopinae**					
*Apoeciluscynicus* (Say, 1831)		e US	13–20	Pr	Phillips 1983
*Euthyrhynchusfloridanus* (Linnaeus, 1767)*		e US to SA	12.0–17.0	Pr	Henry and Froeschner 1988; Thomas 1992
*Perillusbioculatus* (Fabricius, 1775)	x	NA	8.5–11.5	Pr	Thomas 1992
*Perillusstrigipes* (Herrich-Schäffer, 1853)		e US	7.5–10.0	Pr	Thomas 1992
*Podisusbrevispinus* Thomas, 1992		CAN to n US	8.0–11.0	Pr	Phillips 1983; Thomas 1992
*Podisusmaculiventris* (Say, 1831)	x	NA	8.5–13.0	Pr	Furth 1974; Thomas 1992
*Podisusserieventris* (Uhler, 1871)		CAN to US	8.0–11.5	Pr	Thomas 1992; Henry and Froeschner 1988
*Stiretrusanchorago* (Say, 1828)		se CAN to CA	7.0–10.0	Pr	McPherson 1982; Paiero et al. 2013
**Pentatominae**					
**Aelini**					
*Aeliaamericana* Dallas, 1851*		w CAN and US	7.0–9.0	Ph	Henry and Froeschner 1988
*Neottiglossacavifrons* Stål 1872		s US	4.0–5.2	Ph	Rider 1989
*Neottiglossasulcifrons* Stål, 1872		s US	4.0–5.0	Ph	Rider 1989
*Neottiglossaundata* (Say, 1832)*		s CAN and n US	4.5–6.0	Ph	Rider 1989
**Cappaeini**					
*Halyomorphahalys* (Stål, 1855)	x	Cosmopolitan	12.0–17.0	Ph	Rider et al. 2002
**Carpocorini**					
*Coenusdelius* (Say, 1832)*		s CAN and US	8.5–10.5	Ph	Rider 1996
*Cosmopeplalintneriana* (Kirkaldy, 1909)		NA	4.0–7.0	Ph	McDonald 1986
*Euschistuspolitus* Uhler, 1897*		e US	8.2–10.0	Ph	Henry and Froeschner 1988; Paim et al. 2022
*Euschistusservus* (Say, 1832)	x	NA to CA	10.0–15.0	Ph, FaP	Rolston 1974; Paim et al. 2022
*Euschistustristigmus* (Say, 1832)	x	e CAN to CA	8.0–12.0	Ph, FaP	Rolston 1974; Paim et al. 2022
*Euschistusvariolarius* (Palisot de Beauvois, 1805)	x	se CAN to n MX	11.0–15.0	Ph, FaP	Rolston 1974; Paim et al. 2022
*Holcostethuslimbolarius* (Stål, 1872)*		NA	7.0–9.0	Ph	McDonald 1974
*Hymenarcysnervosa* (Say, 1832)		e US to n MX	8.5–11.5	Ph	Rolston 1973
*Mcphersonarcysaequalis* (Say, 1832)		e US to MX	6.0–8.5	Ph	Thomas 2012
*Meneclesinsertus* (Say, 1832)		s CAN to n MEX	12.0–14.0	Ph, FaP	Rolston 1972
*Mormidealugens* (Fabricius, 1775)		e CAN to ne MX	5.0–7.2	Ph	Rolston 1978
*Oebaluspugnax* (Fabricius, 1775)	x	e US to MX	8.0–12.0	Ph, FaP	Sailer 1957
*Proxyspunctulatus* (Palisot de Beauvois, 1818)*		e US to SA	11.0–13.0	Ph, FaP	Rider and Chapin 1992
*Trichopeplasemivittata* (Say, 1832)*		se CAN to MX	5.5–8.0	Ph	McDonald 1976
**Halyini**					
*Brochymenacariosa* Stål, 1872		e US and ne MX	15.8–19.3	Ph	Larivière 1992
*Brochymenaquadripustulata* (Fabricius, 1775)		NA to CA	12.0–18.6	Ph	Henry and Froeschner 1988; McPherson and Ahmad 2007
*Parabrochymenaarborea* (Say, 1825)		se CAN to CA	10.0–18.0	Ph, FaP	McPherson 1982
*Parabrochymenapunctatapunctata* Van Duzee, 1909		se US	14.0–17.0	Ph	Larivière 1992
**Nezarini**					
*Chinaviahilaris* (Say, 1832)	x	NA	13.0–19.0	Ph, Pr	McPherson 1982
*Nezaraviridula* (Linnaeus, 1758)*	x	Cosmopolitan	14.0–17.0	Ph	Ferrari et al. 2010
*Thyantacalceata* (Say, 1832)		e US	7.0–10.5	Ph	Rider and Chapin 1992
*Thyantacustatoraccerra* McAtee, 1919		s CAN to n MEX	9.0–13.0	Ph	Rider and Chapin 1992
**Pentatomini**					
*Banasacalva* (Say, 1832)		NA to CA	8.5–12.0	Ph	Thomas and Yonke 1981
*Banasadimidiata* (Say, 1832)*		NA to CA	8.5–11.0	Ph	Thomas and Yonke 1981
*Banasaeuchlora* (Stål, 1872)*		NA to CA	9.0–11.0	Ph	Thomas and Yonke 1981
*Banasasordida* (Uhler, 1871)		s CAN to n MEX	10.0–11.5	Ph	Thomas and Yonke 1981
**Procleticini**					
*Dendrocorishumeralis* (Uhler, 1877)*		se CAN to MX	6.0–8.5	Ph, FaP	Henry and Froeschner 1988
**Strachiini**					
*Murgantiahistrionica* (Hahn, 1834)	x	NA to CA	8.0–11.5	Ph	McPherson 1982
**Podopinae**					
*Amaurochrouscinctipes* (Say, 1828)*		se CAN and e US	5.0–7.5	Ph	Barber and Sailer 1953

**Figure 1.**
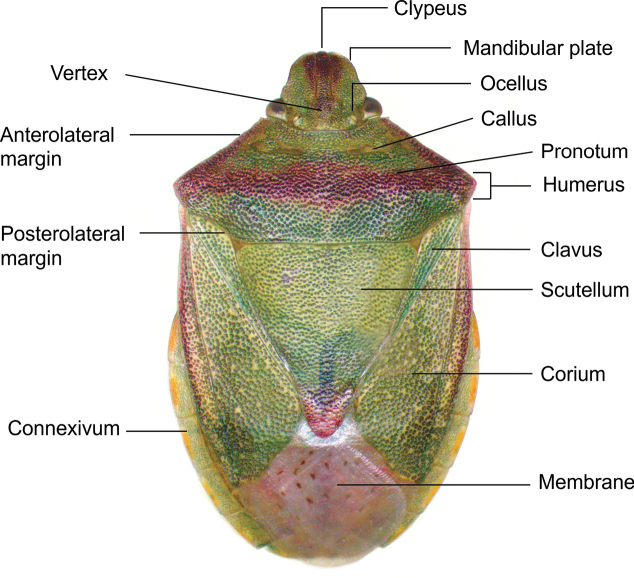
Key morphological features used to identify Pentatomidae (dorsal view).

**Figure 2.**
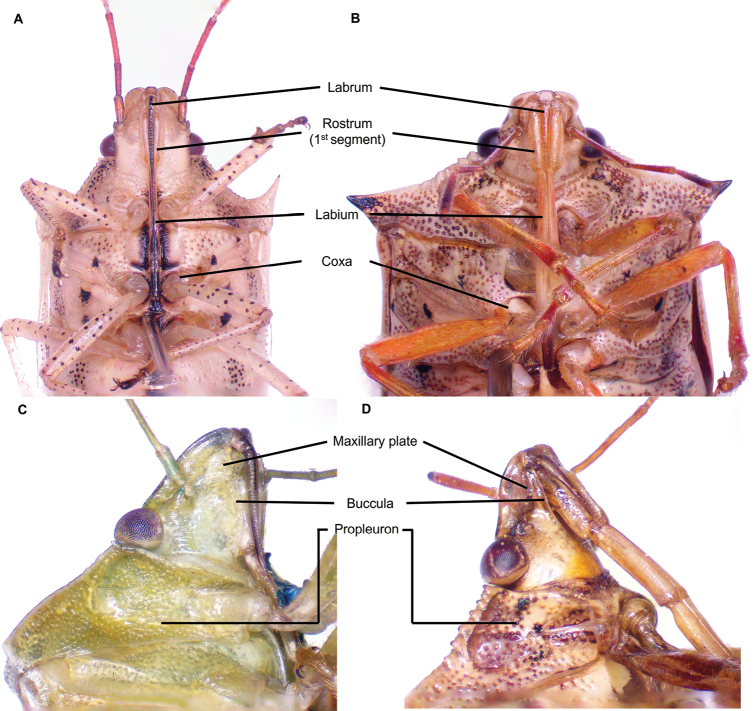
Key morphological features used to identify Pentatomidae (ventral view). Herbivore (**A–C**) and predatory stink bug (**B–D**).

**Plate 1.**
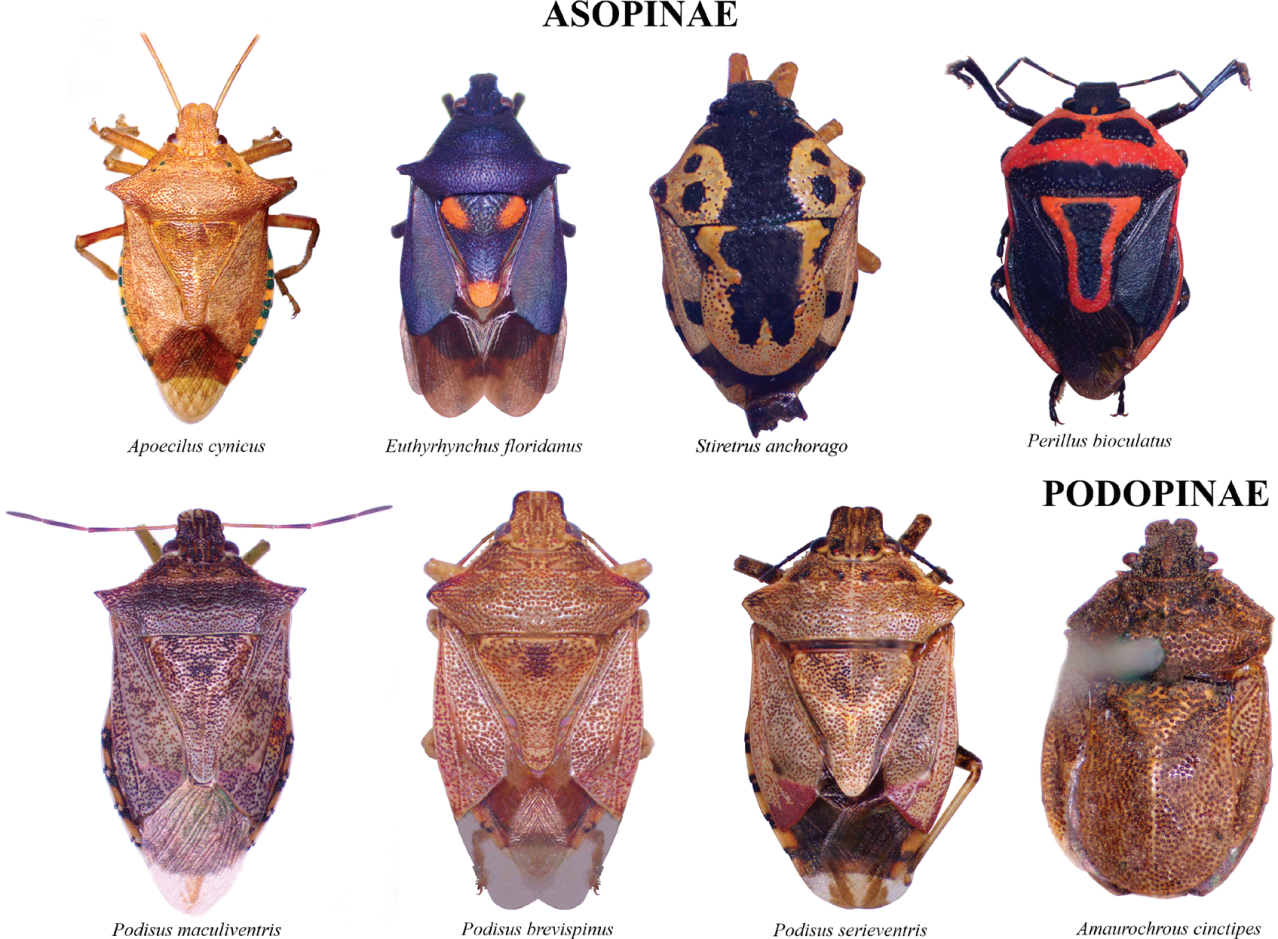
Asopinae and Podopinae.

**Plate 2.**
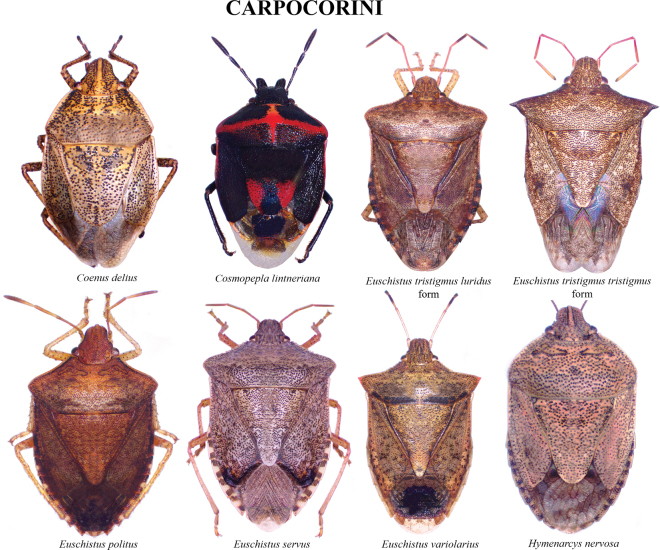
Carpocorini.

**Plate 3.**
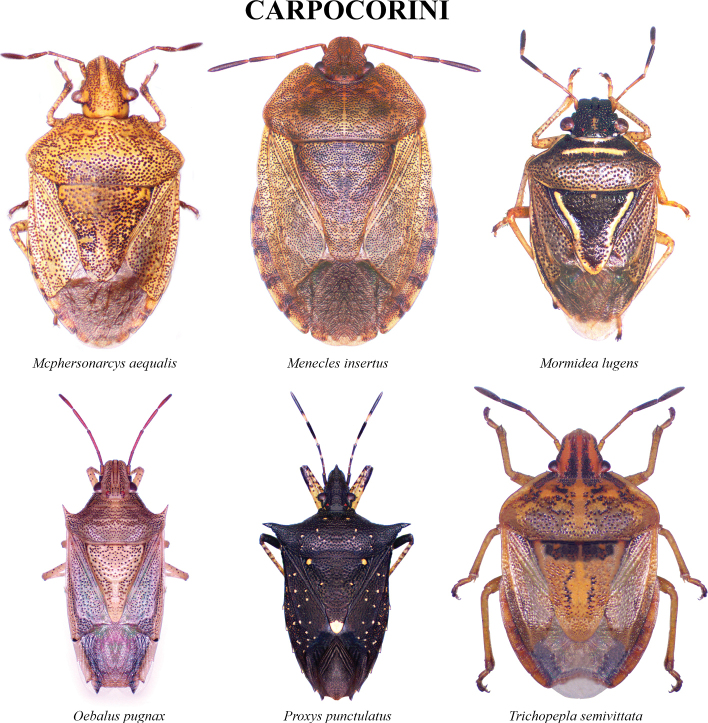
Carpocorini.

**Plate 4.**
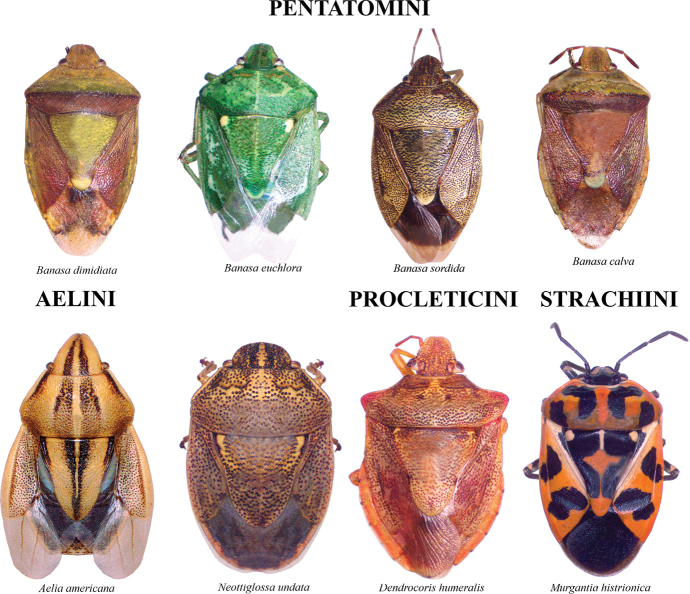
Pentatomini, Aelini, Procleticini, Strachiini.

**Plate 5.**
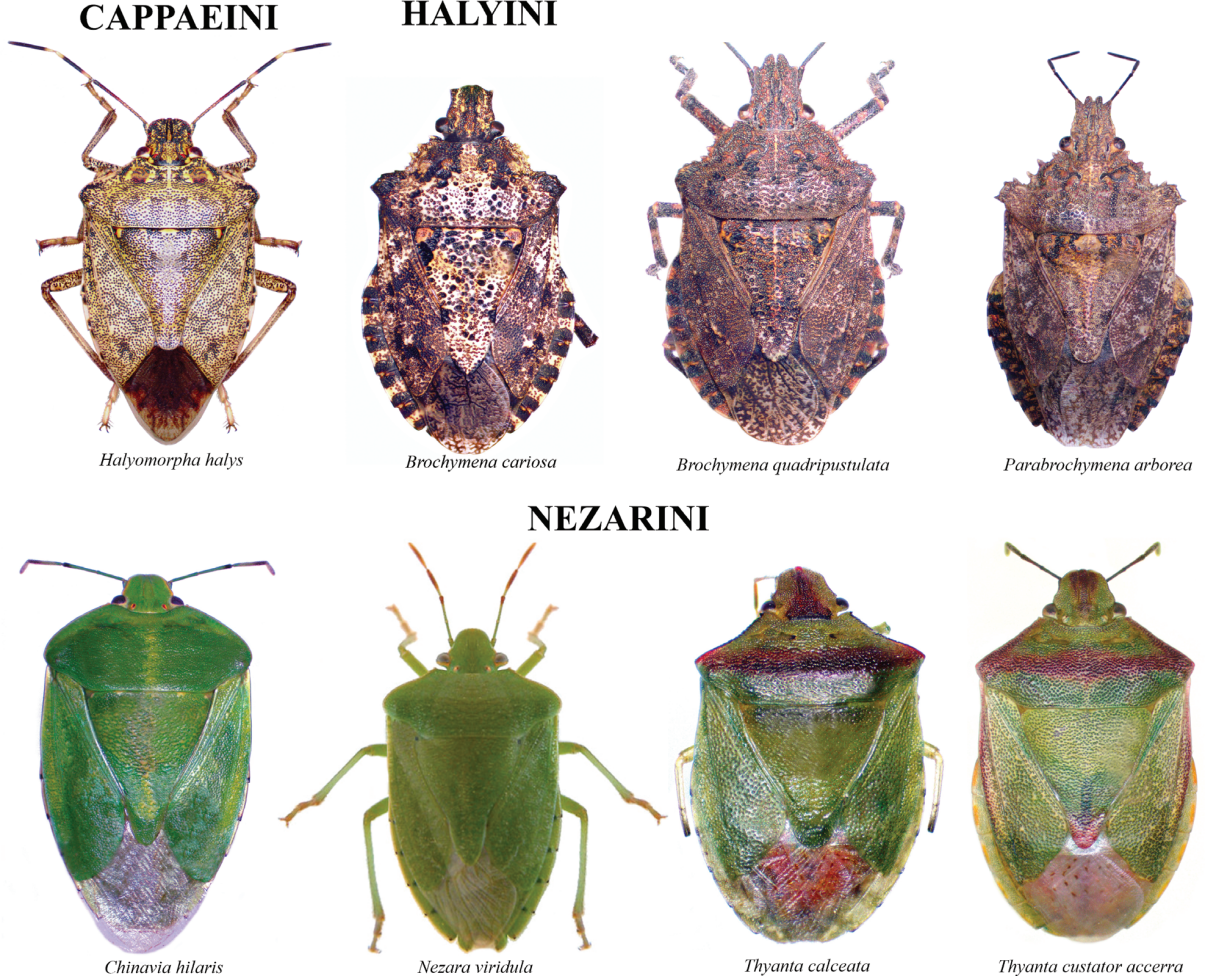
Cappaeini, Halyini, Nezarini.

The species checklist was ordered alphabetically, summarizing information about the distribution, size, feeding habits, and economic importance. The taxonomic catalog used in this study follows the distribution, size, and ecology listed by McPherson (1982) and the synonyms provided by Henry and Froeschner (1988), and Rider and Swanson (2021). The economic importance of species was based on the criteria of Schaefer and Panizzi (2000). Pictures of specimens were taken using an AmScope 18MP camera mounted on a Leica S6D stereoscopic microscope. Images were stacked and cleaned with Adobe PHOTOSHOP v. 22.4.3. Images of 37 species reported in this study are presented in Plates 1–5, as not all the specimens were found in the visited collections. The distribution map and plot were computed in R v. 74.3.1. (R Core Team 2023). Species determinations were conducted following Furth (1974), Larivière (1992), Thomas and Yonke (1981), McPherson (1982), Paiero et al. (2013), Packauskas (2012), and Swanson (2012). The morphological terminology followed Schuh and Weirauch (2020) and Kment and Vilímová (2010) (Figs 1, 2). The occurrence database can be found in the Suppl. material 1.

## ﻿Results

**Figure 3.**
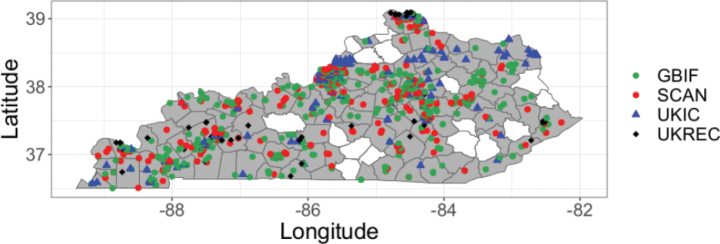
Distribution and source of records of Pentatomidae across Kentucky. Hollow counties lack occurrence records. GBIF = Global Biodiversity Information Facility, SCAN = Symbiota Collections of Arthropods Network, UKIC= University of Kentucky insect collection, UKREC= University of Kentucky’s Research and Education Center.

**Figure 4.**
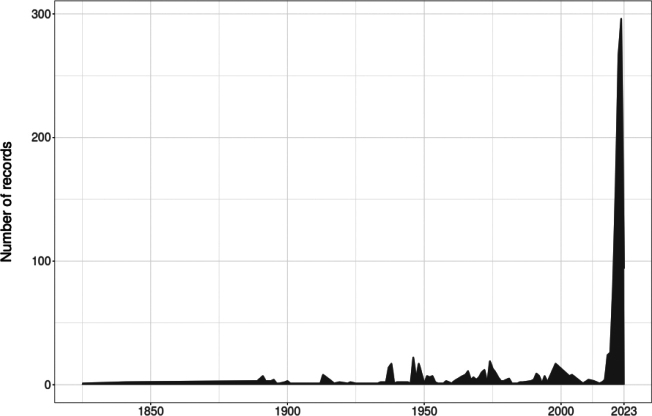
Historical records of Pentatomidae in Kentucky across time.

Overall, the family Pentatomidae is represented by 42 species in 28 genera and 3 subfamilies (Asopinae, Podopinae, and Pentatominae) in Kentucky. Pictures of all species are displayed in Plates 1–5. Thirteen species are new records for Kentucky, 10 species are considered to be of economic importance and seven are strict predators. Nine species are presumably facultative predators (Table 1). *Chinaviahilaris* (Say) and *Halyomorphahalys* were commonly recorded (53%) and 12 species were found as singletons and doubletons. Out of the 1837 records found in Kentucky, 41.3% were obtained from GBIF and 30.3% from SCAN, 22.3% from the UKIC, and 6.1% from the UKREC (Fig. 3). Most records were found around the Louisville and Lexington areas, and 13% of the counties did not have any records of pentatomids: Grayson (West region), Washington, Henry, Owen, Trimble, Gallatin, Montgomery (Central), Lawrence, Martin, Knott, Clay, Leslie, Owsley, Green, Adair, Russell, Cumberland (East) (Fig. 3). Historically, the number of records in Kentucky has been within the order of 20 observations per year. However, observation records remarkably started to increase in 2019 (Fig. 4).

### ﻿Key to species of Pentatomidae of Kentucky

**Table:** 

1	Eyes pedunculate; scutellum U-shaped, enlarged, covering hemelytral membrane	**(subfamily Podopinae) *Amaurochrouscinctipes* Say**
–	Eyes not pedunculate; scutellum either U-shaped or triangular	**2**
2	Rostrum thickened, always directed away from the head; first segment short, thick, never held to the thoracic venter nor contained between the bucculae (Predatory species)	**(subfamily Asopinae) 35**
–	Rostrum not thickened; first segment slender, lying between the bucculae	**(subfamily Pentatominae) 3**
3	Mandibular plates with subapical tooth; pronotum with anterolateral margins coarsely dentate	**4**
–	Mandibular plates without subapical tooth; pronotum smooth or crenulate but never strongly dentate	**7**
4	Basal fourth of scutellum distinctly elevated above the remainder (observed in lateral view); humeri subquadrate	***Parabrochymenaarborea* Say**
–	Basal fourth of scutellum not distinctly elevated above the remainder; humeri subtriangular	**5**
5	Mandibular plates distinctly longer than clypeus and usually converging before clypeus	***Brochymenaquadripustulata* Fabricius**
–	Mandibular plates equal or slightly longer than clypeus; never converging before clypeus	**6**
6	Head appearing roundly truncate anterior to subapical teeth; body greyish white to pale yellowish brown	***Brochymenapunctatapunctata* Van Duzee**
–	Head appearing triangular anterior to subapical teeth; body strikingly mottled with ivory	***Brochymenacariosa* Stål**
7	Abdominal sternite 3 (second visible) medially armed with spine or tubercle	**8**
–	Abdominal sternite 3 (second visible) medially unarmed	**14**
8	Mandibular plates longer than clypeus and converging in front of it; humeri often outlined in red	***Dendrocorishumeralis* Uhler**
–	Mandibular plates not surpassing clypeus, but if so, not converging in front of it; humeri variable	**9**
9	Large species (> 13 mm long), color entirely green dorsally, except for a few black or pale markings; spine on sternite 3 variable	**10**
–	Small species (< 13 mm long), dorsal color different; spine on sternite 3 obtuse	**11**
10	Peritreme long, extending laterally; spine on sternite 3 acute	***Chinaviahilaris* Say**
–	Peritreme short, not extending laterally; spine on sternite 3 obtuse	***Nezaraviridula* Linnaeus**
11	Pronotum with anterior area strongly contrasting with darker color of posterior area	**12**
–	Pronotum with anterior area concolorous with posterior area	**13**
12	Scutellum uniformly brown color, except for the pale green integument at apex	***Banasacalva* Say**
–	Scutellum with anterior half and apex green, posterior lateral margins brown	***Banasadimidiata* Say**
13	Dorsal color green with distinct pale markings irregularly spread throughout; anterior angles of pronotum with conspicuous ivory-white spots	***Banasaeuchlora* Stål**
–	General color brown dorsally; abdominal venter with four rows of dark spots	***Banasasordida* Uhler**
14	Color predominantly black with red, yellow, or white markings	**15**
–	Color predominantly brown or green with variable markings	**17**
15	Small species (< 7 mm); pronotum with red cross; apex of scutellum with 2 red dots	***Cosmopeplalintneriana* Kirkaldy**
–	Large species (> 8 mm), different marking pattern	**16**
16	Color black and orange; humeri rounded, without spines	***Murgantiahistrionica* Hahn**
–	Color black, apex of scutellum white; humeri with sharp spines directed laterally	***Proxyspunctulatus* Palisot de Beauvois**
17	Scutellum equal to or longer than corium	**18**
–	Scutellum shorter than corium	**22**
18	Clypeus distinctly elevated above mandibular plates	***Coenusdelius* Say**
–	Clypeus scarcely elevated above mandibular plates	**19**
19	Prostethium with anterior margin extending beyond anterior margin of eye; costal margin of coria distinctly paler than the inner area	***Aeliaamericana* Dallas**
–	Prostethium, if present, with anterior margin not reaching anterior margin of eye; coria concolorous	**20**
20	Dorsal surface of head and propleura with pale yellow-brown areas; clypeus with median yellow line	***Neottiglossaundata* Say**
–	Head and propleura different	**21**
21	Apex of head broadly rounded, dorsal surface of head deeply concave and covered with short inward-curving hairs	***Neottiglossacavifrons* Stål**
–	Apex of head more tapering, narrowly rounded; dorsal surface of head not distinctly concave and without short hairs	***Neottiglossasulcifrons* Stål**
22	Clypeus distinctly elevated above mandibular plates	***Mcphersonarcysaequalis* Say**
–	Clypeus scarcely elevated above mandibular plates	**23**
23	Base of antennal segment 5 and apex and base of segment 4 pale; venter of head and thorax with clusters of dark (metallic green under bright light) punctures	***Halyomorphahalys* Stål**
–	Antennae color without pale bands; body punctures without metallic reflections	**24**
24	Humeri with sharp spine directed anteriorly	***Oebaluspugnax* Fabricius**
–	Humeri unarmed, if spines present, then not projecting anteriorly	**25**
25	Anterolateral margin of pronotum crenulate	**26**
–	Anterolateral margin of pronotum not crenulate	**29**
26	Abdominal sterna with 1–4 median black spots, which are occasionally reduced or obsolete	***Euschistustristigmus* Say**
–	Abdominal sterna immaculate, if black spot present, then only on male pygophore	**27**
27	Small species (< 10 mm); anterolateral margins of pronotum weakly crenulate; posterior margin of pygophore with V-shaped excavation medially	***Euschistuspolitus* Uhler**
–	Larger species (> 10 mm); anterolateral margins of pronotum distinctly crenulate; posterior margin of pygophore not notched or excavated	**28**
28	Mandibular plates equal or subequal in length to clypeus; antennae with segment five and apical half of segment four black; humeri acute to spinose; male pygophore with a medial black spot	***Euschistusvariolarius* Palisot de Beauvois**
–	Mandibular plates distinctly longer than clypeus; antennae not as above; humeri obtuse; pygophore without markings	***Euschistusservus* Say**
29	Pronotum with margins explanate, projecting forward to eye; pronotum and anterior half of scutellum with pale longitudinal ridge along middle	***Meneclesinsertus* Say**
–	Without the combination above; margins of pronotum not projecting forward to eye	**30**
30	Anterolateral pronotal margins arcuate; hemelytral membrane with veins anastomosing	***Hymenarcysnervosa* Say**
–	Anterolateral pronotal margins not arcuate; hemelytral membrane with veins not anastomosing	**31**
31	Scutellum completely black, ivory color on lateral margins; pronotum with 1 ivory transversal stripe behind anterior margin	***Mormidealugens* Fabricius**
–	Scutellum at most only with black markings; pronotum without transversal stripes	**32**
32	Mandibular plates longer than clypeus by distance equal to at least width of clypeus apex	***Holcostethuslimbolarius* Stål**
–	Mandibular plates equal to or slightly longer than clypeus	**33**
33	Body distinctly covered with fine pubescence	***Trichopeplasemivittata* Say**
–	Body distinctly bare, without distinct pubescence	**34**
34	Anterolateral pronotal margins black	***Thyantacalceata* Say**
–	Anterolateral pronotal margins not black	***Thyantacustatoraccerra* McAtee**
35	Anterior femora armed with ventral spine or tubercle at distal third to fourth	**36**
–	Anterior femora unarmed	**38**
36	Scutellum U-shaped, broadly rounded apically and almost reaching tip of abdomen	***Stiretrusanchorago* Say**
–	Scutellum not U-shaped, nor rounded; never reaching tip of abdomen	**38**
37	Anterior femora with tubercle obsolescent; pronotum with yellow or orange longitudinal stripe on midline	***Perillusstrigipes* Herrich-Schäffer**
–	Anterior femora with stout tubercle or spine; color markings variable (white, yellow or red)	***Perillusbioculatus* Fabricius**
38	Rostrum reaching abdominal sternite 3; abdominal sternite 3 medially unarmed	***Euthyrhynchusfloridanus* Linnaeus**
–	Rostrum not reaching abdominal sternite 3; abdominal sternite 3 medially with distinct spine	**39**
39	Mandibular plates longer than clypeus; large species (> 14 mm)	***Apoeciluscynicus* Say**
–	Mandibular plates equal than clypeus; small species (< 12 mm)	**40**
40	Humeri produced into outward-projecting spines; spine on sternite 3 long, reaching anterior margin of hind coxae	***Podisusmaculiventris* Say**
–	Humeri blunt, not produced into spines; spine on sternite 3 variable, but not reaching anterior margin of hind coxae	**41**
41	Hind femora almost immaculate; spine on sternite 3 short, not reaching hind coxae	***Podisusbrevispinus* Thomas**
–	Hind femora heavily covered with black spots; spine on sternite 3 reaching posterior margin of hind coxae	***Podisusserieventris* Uhler**

## ﻿Discussion

The taxonomy of the family Pentatomidae is known to be entangled due to the large diversity and challenging species complexes, which has led to copious attempts to solve these issues over the last decades. Overall, the taxonomy of stink bugs in North America is well developed but outdated; thus, regional keys have become relevant for understanding the distribution, conservation, and management of species in agricultural systems (Packauskas 2012; Paiero et al. 2013; Koch et al. 2014, 2017; Pezzini et al. 2019). In this work, the first list and key to species occurring in Kentucky are presented. Furthermore, pictures of all species are provided for comparison and identification purposes. This becomes valuable to extension educators, students, and non-specialists, especially since some species can be easily confused without taxonomic training or available keys.

Our knowledge of the pentatomid fauna of Kentucky significantly increased after this work (42 species of which 13 are new records); which also provides insights into species distributions (see Table 1). *Apoeciluscynicus*, *Brochymenacariosa*, *Euschistuspolitus*, *Hymenarcysnervosa*, *Mcphersonarcysaequalis*, *Oebaluspugnax*, and *Thyantacalceata* are restricted to the eastern United States north of Mexico. Other species, such as *Perillusstrigipes* Herrich-Schäffer, *Neottiglossasulcifrons* Stål, *N.cavifrons* Stål, *Brochymenapunctatapunctata* Van Duzee, were recorded in Kentucky more than three decades ago (Froeschner 1988; Furth 1974; Larivière 1992, respectively); however, they were not found in the visited collections or public databases. Perhaps future works could corroborate the presence of these species in Kentucky and provide proper photographs.

It was not surprising to find the brown marmorated stink bug, *Halyomorphahalys* as the most commonly recorded species in Kentucky (30%), as this invasive species (originally from East Asia) is well established in the eastern United States (Lee et al. 2013). The brown marmorated stink bug is known for its impact on agriculture as it severely damages tree fruit and other crops in North America (Leskey and Nielsen 2018). Besides *H.halys*, we found eight stink bug species occurring in Kentucky that are considered pests in agricultural systems. All of them are widespread across North America and some (3 species) occur all the way down to Central America. *Nezaraviridula* was not recorded in Kentucky before this work. This cosmopolitan species, referred to as the southern green stink bug, has long been considered a key pest in the tropical and subtropical regions worldwide (especially attacking *Glycinemax* L. Merrill), between latitudes 45°N and 45°S, and it is still spreading to new areas (Todd 1989). Although formerly abundant populations have been declining in the last 15 years in warmer areas of the Americas, presumably given the combination of several factors, i.e., climate change, parasitism, competition, weed control, and cropping systems (Panizzi and Lucini 2016).

Even though many species found in Kentucky are phytophagous, 25% exhibit predaceous habits and 17.5% are strict predators (subfamily Asopinae). Predatory stink bugs play a key role in natural and agricultural habitats since they control the population of arthropods (Richman and Whitcomb 1978). There are about 300 species of Asopinae described worldwide that are generalist predators mainly feeding on slow-moving, soft-bodied insects, primarily larval forms of the Lepidoptera, Coleoptera, and Hymenoptera (De Clercq 2008). *Podisusmaculiventris* was the most common predacious species recorded in Kentucky (1.8% of records). This species has been widely studied in North America since it is usually found in agricultural systems (Mukerji and LeRoux 1969; Wiedenmann and O’Neil 1991; Linder et al. 2023). In Kentucky, it is not rare to find *P.maculiventris* in field crops during the growing season, but it could be mistaken for *Euschistus* species without a trained eye.

Occurrence data of stink bugs in Kentucky provided important insights into the understanding of sampling bias and gaps. In this case, several counties in the central and eastern regions do not have records of pentatomids, namely Grayson (West region), Washington, Henry, Owen, Trimble, Gallatin, Montgomery (Central), Lawrence, Martin, Knott, Clay, Leslie, Owsley, Green, Adair, Russell, Cumberland (East). Most observations/collects were found around highly populated areas in Kentucky i.e., Louisville and Lexington (U.S. Census Bureau 2024). Insect sampling is typically influenced by cities and roads, which are known as handy locations to take samples or simply take pictures (Falcon-Brindis et al. 2021). The exponential increase in observations starting in 2019 can be attributed to the active participation of citizens on insect identification forums such as iNaturalist. In fact, 92% of the records downloaded from GBIF were research-grade observations from this open-source platform.

## ﻿Conclusion

In this study, we provided an up-to-date list of stink bugs (Pentatomidae) found in Kentucky, a dichotomous key of stink bugs, and high-quality pictures of all species. Overall, the family Pentatomidae is represented by 42 species in 28 genera and three subfamilies (Asopinae, Podopinae, and Pentatominae). This study establishes a baseline of the knowledge of stink bug fauna and will leverage the integrated pest management programs needing monitoring and identification of native and exotic species. This work also summarizes the distribution, size, and economic importance of the Pentatomidae species occurring in Kentucky. Both preserved specimens and public records of stink bugs greatly contributed to the understanding of sampling efforts and biases (i.e., towards populated areas).

## ﻿Additional information

### Author ORCIDs

Armando Falcon-Brindis https://orcid.org/0000-0003-2496-2178

### Data availability

All of the data that support the findings of this study are available in the main text or Supplementary Information.

## Supplementary materials

Supplementary material 1 Occurrence database Data type csv https://binary.pensoft.net/file/1141165This dataset is made available under the Open Database License (http://opendatacommons.org/licenses/odbl/1.0/). The Open Database License (ODbL) is a license agreement intended to allow users to freely share, modify, and use this Dataset while maintaining this same freedom for others, provided that the original source and author(s) are credited.Armando Falcon-Brindis

## References

[B1] Barber HG Sailer RI (1953) A revision of the turtle bugs of North America (Hemiptera: Pentatomidae). Journal of the Washington Academy of Sciences 43(5): 150–162

[B2] Brugnera R Limberger GM Campos LA Grazia J (2022) The eggs and nymphs of predatory stink bugs (Hemiptera: Pentatomidae: Asopinae): what do we know? Zoology 151: 125991. 10.1016/j.zool.2021.125991 35257984

[B3] Bundy CS (2012) An annotated checklist of the stink bugs (Heteroptera: Pentatomidae) of New Mexico. Great Lakes Entomologist 45(3–4): 196–209. 10.22543/0090-0222.2252

[B4] Cassis G Schuh RT (2012) Systematics, Biodiversity, Biogeography, and Host Associations of the Miridae (Insecta: Hemiptera: Heteroptera: Cimicomorpha). Annual Review of Entomology 57: 377–404. 10.1146/annurev-ento-121510-133533 22149267

[B5] Chapman AD (2005) Principles and Methods of Data Cleaning: Primary Species and Species-Occurrence Data, version 1.0. Report for the Global Biodiversity Information Facility, Copenhagen. http://www.gbif.org/document/80528

[B6] De Clercq PD (2008) Stink Bugs, Predatory (Hemiptera: Pentatomidae, Asopinae). In: Capinera JL (Ed.) Encyclopedia of Entomology.Springer, Dordrecht, 2122–2125. 10.1007/978-1-4020-6359-6_3115

[B7] DeCoursey RM Allen RC (1968) A generic key to the nymphs of the Pentatomidae of the Eastern United States (Hemiptera: Heteroptera). University of Connecticut Occasional Papers 1: 141–151.

[B8] Falcon-Brindis A León-Cortés JL Montañez-Reyna M (2021) How effective are conservation areas to preserve biodiversity in Mexico? Perspectives in Ecology and Conservation 19(4): 399–410. 10.1016/j.pecon.2021.07.007

[B9] Ferrari A Schwertner CF Grazia J (2010) Review, cladistic analysis and biogeography of *Nezara* Amyot & Serville (Hemiptera: Pentatomidae). Zootaxa 2024(1): 1–41. 10.11646/zootaxa.5022.1.1

[B10] Froeschner RC (1988) Family Pentatomidae Leach, 1815. The Stink Bugs. In: Henry TJ Froeschner RC (Eds) Catalog of the Heteroptera, or true bugs, of Canada and the continental United States.E. J. Brill, New York, 544–597. 10.1163/9789004590601_032

[B11] Furth DG (1974) The stink bugs of Ohio (Hemiptera: Pentatomidae). Bulletin of the Ohio Biological Survey 5(1): 1–62.

[B12] GBIF.org (2023) GBIF Home Page. https://www.gbif.org [22 August 2023]

[B13] Grazia J Panizzi AR Greve C Schwertner CF Campos LA Garbelotto TA Fernandes JAM (2015) Stink Bugs (Pentatomidae). In: Panizzi AR, Grazia J (Eds) True Bugs (Heteroptera) of the Neotropics, Springer, New York, 901 pp. 10.1007/978-94-017-9861-7

[B14] Henry TJ Froeschner RC (1988) Catalog of the Heteroptera, or True Bugs, of Canada and the Continental United States. E. J. Brill, Leiden, New York, 958 pp.

[B15] Henry TJ Froeschner RC (1992) Corrections and additions to the “Catalog of the Heteroptera, or true bugs, of Canada and the Continental United States.” Proceedings of the Entomological Society of Washington 94(2): 263–272.

[B16] Herring JL Ashlock PD (1971) A Key to the Nymphs of the Families of Hemiptera (Heteroptera) of America North of Mexico. The Florida Entomologist 54(3): 207–212. 10.2307/3493715

[B17] Hoebeke ER Carter ME (2003) *Halyomorphahalys* (Stål) (Heteroptera: Pentatomidae): A polyphagous plant pest from Asia newly detected in North America. Proceedings of the Entomological Society of Washington 105: 225–237.

[B18] Hoffman RL (1971) The Insects of Virginia: No. 4. Shield bugs (Hemiptera; Scutelleroidea, Corimelaenidae, Cydnidae, Pentatomidae). Virginia Polytechnic Institute and State University, Research Division Bulletin 67, 61 pp.

[B19] Kment P Vilímová J (2010) Thoracic scent efferent system of Pentatomoidea (Hemiptera: Heteroptera): a review of terminology. Zootaxa 2706: 1–77. 10.11646/zootaxa.2706.1.1

[B20] Koch RL Rider DA Tinerella PP Rich WA (2014) Stink Bugs (Hemiptera: Heteroptera: Pentatomidae) of Minnesota: An Annotated Checklist and New State Records. The Great Lakes Entomologist 47(3–4): 171–185. 10.22543/0090-0222.2305

[B21] Koch RL Pezzini DT Michel AP Hunt TE (2017) Identification, biology, impacts, and management of stink bugs (Hemiptera: Heteroptera: Pentatomidae) of soybean and corn in the Midwestern United States. Journal of Integrated Pest Management 8(1): 1–14. 10.1093/jipm/pmx004

[B22] Larivière MC (1992) Description of *Parabrochymena*, new genus, and redefinition and review of *Brochymena* Amyot and Audinet-Serville (Hemiptera: Pentatomidae), with considerations on natural history, chorological affinities, and evolutionary relationships. Memoirs of the Entomological Society of Canada 163: 1–75. 10.4039/entm124163fv

[B23] Lee DH Short BD Joseph SV Bergh JC Leskey TC (2013) Review of the Biology, Ecology, and Management of *Halyomorphahalys* (Hemiptera: Pentatomidae) in China, Japan, and the Republic of Korea. Environmental Entomology 42(4): 627–641. 10.1603/EN13006 23905725

[B24] Leskey TC Nielsen AL (2018) Impact of the invasive brown marmorated stink bug in North America and Europe: history, biology, ecology, and management. Annual Review of Entomology 63: 599–618. 10.1146/annurev-ento-020117-043226 29068708

[B25] Linder S Jarrett BJ Szűcs M (2023) Non-target attack of the native stink bug, *Podisusmaculiventris* by *Trissolcusjaponicus*, comes with fitness costs and trade-offs. Biological Control 177: 105107. 10.1016/j.biocontrol.2022.105107

[B26] McDonald FJD (1974) Revision of the Genus *Holcostethus* in North America (Hemiptera: Pentatomidae). Journal of the New York Entomological Society 82(4): 245–258.

[B27] McDonald FJD (1976) Revision of the genus *Trichopepla* (Hemiptera:Pentatomidae) in N. America. Journal of the New York Entomological Society 84(1): 9–22

[B28] McDonald FJD (1986) Revision of *Cosmopepla* Stål (Hemiptera: Pentatomidae). Journal of the New York Entomological Society 94: 1–15.

[B29] McPherson JE (1982) The Pentatomoidea (Hemiptera) of Northeastern North America with emphasis on the fauna of Illinois. Southern Illinois University Press, Carbondale, Illinois, 240 pp.

[B30] McPherson JE (2018) Invasive stink bugs and related species (Pentatomoidea): biology, higher systematics, semiochemistry, and management. CRC Press, Taylor & Francis Group, Boca Raton, Florida, 819 pp. 10.1201/9781315371221

[B31] McPherson JE Ahmad I (2007) Redescriptions of *Brochymena* and *Parabrochymena* (Hemiptera: Heteroptera: Pentatomidae), based primarily on male genitalia, with reclassiﬁcation of three species and description of New World tribe (Halyini). Annals of the Entomological Society of America 100(5): 673–682. 10.1603/0013-8746(2007)100[673:ROBAPH]2.0.CO;2

[B32] McPherson JE McPherson RM (2000) Stink bugs of economic importance in America north of Mexico. CRC Press, Boca Raton, 253 pp. 10.1201/9781420042429

[B33] Mitchell PL Zeilinger AR Medrano EG Esquivel JF (2018) Pentatomids as vectors of plant pathogens. In: McPherson JE (Ed.) Invasive stink bugs and related species (Pentatomoidea): biology, higher systematics, semiochemistry, and management.CRC Press, Taylor & Francis Group, Boca Raton, Florida, 611–640. 10.1201/9781315371221-13

[B34] Mukerji MK LeRoux EJ (1969) A quantitative study of food consumption and growth of *Podisusmaculiventris* (Hemiptera: Pentatomidae). The Canadian Entomologist 101(4): 387–403. doi:10.4039/Ent101387-4

[B35] O’Donnell JE Schaefer CW (2012) Annotated checklist of the Pentatomidae (Heteroptera) of Connecticut. Great Lakes Entomologist 45(3–4): 220–234. 10.22543/0090-0222.2254

[B36] Packauskas RJ (2012) The Pentatomidae, or Stink Bugs, of Kansas with a key to species (Hemiptera: Heteroptera). The Great Lakes Entomologist 45(3–4): 210–219. 10.22543/0090-0222.2253

[B37] Paiero SM Marshall SA McPherson JE Ma MS (2013) Stink bugs (Pentatomidae) and parent bugs (Acanthosomatidae) of Ontario and adjacent areas: A key to species and a review of the fauna. Canadian Journal of Arthropod Identification 24: 1–183. 10.3752/cjai.2013.24

[B38] Paim MR Grazia J Rider DA Bianchi FM (2022) Revisiting Stål’s thoughts: formalizing the *ictericus* group in Euschistus (Euschistus) (Hemiptera: Heteroptera: Pentatomidae). Zootaxa 5169(6): 501–537. 10.11646/zootaxa.5169.6.1 36095425

[B39] Panizzi AR Lucini T (2016) What Happened to *Nezaraviridula* (L.) in the Americas? Possible Reasons to Explain Populations Decline. Neotroprical Entomology 45: 619–628. 10.1007/s13744-016-0446-2 27804018

[B40] Panizzi AR Slansky F (1985) Review of phytophagous pentatomids (Hemiptera: Pentatomidae) associated with soybean in the Americas. Florida Entomologist 68: 184–214. 10.2307/3494344

[B41] Panizzi AR McPherson JE James DG Javahery M McPherson RM (2000) Stink Bugs (Pentatomidae). In: Schaefer CW Panizzi AR (Eds) Heteroptera of economic importance.CRC Press, Washington, D.C., 421–474. 10.1201/9781420041859.ch13

[B42] Pezzini DT DiFonzo CD Finke DL Hunt TE Knodel JJ Krupke CH McCornack B Michel AP Philips CR Varenhorst AJ Wright RJ Koch RL (2019) Community composition, abundance, and phenology of stink bugs (Hemiptera: Pentatomidae) in soybean in the North Central Region of the United States. Journal of Economic Entomology 112(4): 1722–1731. 10.1093/jee/toz099 31038171

[B43] Phillips KA (1983) A taxonomic revision of the Nearctic species of *Apateticus* Dallas and *Podisus* Herrich-Schaeffer (Heteroptera: Pentatomidae: Asopinae). Ph. D. Dissertation, Oregon State University, Corvallis, Oregon, 275 pp.

[B44] R Core Team (2023) R: A Language and Environment for Statistical Computing; R Foundation for Statistical Computing: Vienna, Austria. https://www.R-project.org/

[B45] Richman DB Whitcomb WH (1978) Comparative lifecycle of four species of predatory stink bugs. The Florida Entomologist 61(3): 113–119. 10.2307/3494225

[B46] Rider DA (1989) Review of the New World species of the genus *Neottiglossa* Kirby (Heteroptera: Pentatomidae). Journal of the New York Entomological Society 97: 394–408.

[B47] Rider DA (1996) Review of the genus *Coenus* Dallas, with the description of *C.explanatus*, new species (Heteroptera: Pentatomidae). Journal of the New York Entomological Society 103: 39–47.

[B48] Rider DA (2012) The Heteroptera (Hemiptera) of North Dakota I: Pentatomomorpha: Pentatomoidea. The Great Lakes Entomologist 45: 312–380. 10.22543/0090-0222.2258

[B49] Rider DA (2015) Pentatomoidea Home Page. https://www.ndsu.edu/pubweb/~rider/Pentatomoidea/ [12 May 2024]

[B50] Rider DA Chapin JB (1992) Revision of the Genus *Thyanta* Stål, 1862 (Heteroptera: Pentatomidae) II. North America, Central America, and the West Indies. Journal of the New York Entomological Society 100(1): 42–98.

[B51] Rider DA Swanson DR (2021) A distributional synopsis of the Pentatomidae (Heteroptera) north of Mexico, including new state and provincial records. Zootaxa 5015(1): 001–069. 10.11646/zootaxa.5015.1.1 34810466

[B52] Rider DA Zheng LY Kerzhner IM (2002) Checklist and nomenclatural notes on the Chinese Pentatomidae (Heteroptera). II. Pentatominae. Zoosystematica Rossica 11(1): 135–153. 10.31610/zsr/2002.11.1.135

[B53] Rider DA Schwertner CF Vilímová J Rédei D Kment P Thomas DB (2018) Higher systematics of the Pentatomoidea. In: McPherson JE (Ed.) Invasive Stink Bugs and Related Species (Pentatomoidea) Biology, Higher Systematics, Semiochemistry, and Management.CRC Press, Boca Raton, Florida, 25–204. 10.1201/9781315371221-2

[B54] Roca-Cusachs M Schwertner CF Kim J Eger J Grazia J Jung S (2022) Opening Pandora’s box: molecular phylogeny of the stink bugs (Hemiptera: Heteroptera: Pentatomidae) reveals great incongruences in the current classification. Systematic Entomology 47(1): 36–51. 10.1111/syen.12514

[B55] Rolston LH (1972) The genus *Menecles* Stål (Hemiptera; Pentatomidae). Journal of the New York Entomological Society 80(4): 234–237.

[B56] Rolston LH (1973) A review of *Hymenarcys* (Hemiptera: Pentatomidae). Journal of the New York Entomological Society 81: 111–117.

[B57] Rolston LH (1974) Revision of the genus *Euschistus* in middle America (Hemiptera, Pentatomidae, Pentatomini). Entomologia Americana 48: 1–102.

[B58] Rolston LH (1978) A Revision of the Genus *Mormidea* (Hemiptera: Pentatomidae). Journal of the New York Entomological Society 86(3): 161–219.

[B59] Sailer RI (1957) *Solubea* Bergoth 1891, a synonym of *Oebalus* Stal 1862, and a note concerning the distribution of *O.ornatus* (Sailer). Proceedings of the Entomological Society of Washington 59(1): 41–42.

[B60] SCAN (2023) Symbiota Collections of Arthropods Network: A Data Portal Built to Visualize, Manipulate, and Export Species Occurrences. https://scan-bugs.org/portal/ [3 September 2023]

[B61] Schaefer CW Panizzi AR (2000) Heteroptera of economic importance. CRC Press, Washington, D.C., 828 pp. 10.1201/9781420041859

[B62] Schuh RT Weirauch C (2020) True Bugs of the World (Hemiptera: Heteroptera), Classification and Natural History (Second Edition). Siri Scientific Press Monograph Series (Vol. 8). Siri Scientific Press, Manchester, 767 pp.

[B63] Sites RW Simpson KB Wood DL (2012) The stink bugs (Hemiptera: Heteroptera: Pentatomidae) of Missouri. The Great Lakes Entomologist 45: 134–163. 10.22543/0090-0222.2249

[B64] Swanson DR (2012) An Updated Synopsis of the Pentatomoidea (Heteroptera) of Michigan. The Great Lakes Entomologist 45(2): 263–311. 10.22543/0090-0222.2257

[B65] Thomas DB (1992) Taxonomic synopsis of the Asopine Pentatomidae (Heteroptera) of the Western Hemisphere. Lanham, The Thomas Say Foundation, ESA, Monographs, 156 pp. 10.4182/OACO2099

[B66] Thomas DB (2012) *Mcphersonarcys*, a new genus for *Pentatomaaequalis* Say (Heteroptera: Pentatomidae). The Great Lakes Entomologist 45(2): 127–133. 10.22543/0090-0222.2248

[B67] Thomas DB Yonke TR (1981) A Review of the Nearctic species of the genus *Banasa* Stål (Hemiptera: Pentatomidae). Journal of the Kansas Entomological Society 54(2): 233–248.

[B68] Todd JW (1989) Ecology and behavior of *Nezaraviridula*. Annual review of entomology 34(1): 273–292. 10.1146/annurev.en.34.010189.001421

[B69] U. S. Census Bureau (2024) Kentucky QuickFacts. https://data.census.gov/profile/Kentucky?g=040XX00US21 [17 January 2024]

[B70] Waddill V Shepard M (1974) Biology of a predaceous stink bug, *Stiretrusanchorago*, (Hemiptera: Pentatomidae). Florida Entomologist 57: 249–253. 10.2307/3493252

[B71] Weirauch C (2008) Cladistic analysis of Reduviidae (Heteroptera: Cimicomorpha) based on morphological characters. Systematic Entomology 33: 229–274. 10.1111/j.1365-3113.2007.00417.x

[B72] Wiedenmann RN O’Neil RJ (1991) Searching behavior and time budgets of the predator *Podisusmaculiventris*. Entomologia experimentalis et applicata 60(1): 83–93. 10.1111/j.1570-7458.1991.tb01525.x

[B73] Zack RS Landolt PJ Munyaneza JE (2012) The stink bugs (Hemiptera: Heteroptera: Pentatomidae) of Washington state. The Great Lakes Entomologist 45: 251–262. 10.22543/0090-0222.2256

